# Novel RNA Viruses from the Transcriptome of Pheromone Glands in the Pink Bollworm Moth, *Pectinophora gossypiella*

**DOI:** 10.3390/insects12060556

**Published:** 2021-06-15

**Authors:** Xiaoyi Dou, Sijun Liu, Victoria Soroker, Ally Harari, Russell Jurenka

**Affiliations:** 1 Department of Entomology, Iowa State University, Ames, IA 50011, USA; xydou@uga.edu (X.D.); rjurenka@iastate.edu (R.J.); 2 The Volcani Center, Institute of Plant Protection, ARO, Bet-Dagan 50250, Israel; sorokerv@volcani.agri.gov.il (V.S.); aharari@volcani.agri.gov.il (A.H.)

**Keywords:** single-stranded RNA virus, iflavirus, pink bollworm, transcriptome

## Abstract

**Simple Summary:**

The pink bollworm, *Pectinophora gossypiella* (Lepidoptera: Gelechiidae), is a major pest of cotton. In this study, we analyzed the mRNA from pheromone glands of two populations in Israel. We found several virus sequences that were the same in these populations. We identified these viruses based on high-throughput sequencing data and analysis of the assembled transcripts. Through analysis of the sequences, we identified several unique viral sequences representing possible novel viral species. Two of the viral sequences were found in relatively high abundance in pheromone glands. One of the virus sequences was also found through analysis of previous transcriptome sequencing data from the midgut of pink bollworm larvae. This is the first report of these unique viral sequences found in the pink bollworm, and these viruses could be developed to help control this pest around the world, but more research is needed to determine their utility as biological control agents.

**Abstract:**

In this study, we analyzed the transcriptome obtained from the pheromone gland isolated from two Israeli populations of the pink bollworm *Pectinophora gossypiella* to identify viral sequences. The lab population and the field samples carried the same viral sequences. We discovered four novel viruses: two positive-sense single-stranded RNA viruses, Pectinophora gossypiella virus 1 (PecgV1, a virus of *Iflaviridae*) and Pectinophora gossypiella virus 4 (PecgV4, unclassified), and two negative-sense single-stranded RNA viruses, Pectinophora gossypiella virus 2 (PecgV2, a virus of *Phasmaviridae*) and Pectinophora gossypiella virus 3 (PecgV3, a virus of *Phenuiviridae*). In addition, sequences derived from two negative-sense single-stranded RNA viruses that belong to *Mononegavirales* were found in the data. Analysis of previous transcriptome sequencing data derived from the midgut of pink bollworm larvae of a USA population only identified PecgV1, but no other viruses. High viral sequence coverages of PecgV1 and PecgV4 were observed in both field and lab populations. This is the first report of viral sequences discovered from the pink bollworm. Results from this investigation suggest that the pink bollworm harbors multiple viruses. Further investigation of the viral pathogens may help to develop novel pest management strategies for control of the pink bollworm.

## 1. Introduction

The pink bollworm (PBW), *Pectinophora gossypiella* (Lepidoptera: Gelechiidae), is a worldwide pest that causes significant yield losses in cotton fields. The larvae of the pink bollworm feed inside flower buds and bolls, where they are well protected from contact to insecticides; hence, application of traditional insecticides is not efficient in controlling the pest. Current control measures for PBW largely depend on the use of transgenic cotton producing the *Bacillus thuringiensis* (Bt) toxin, which effectively controls this pest [1]. The use of Bt cotton showed a long-term suppression of pink bollworm in a ten-year study in Arizona, USA [1]. However, PBW populations resistant to Bt toxin Cry1Ac have been observed in the field [2]. In addition, artificial sex pheromones used in mating disruption have been applied in many areas. They successfully controlled PBW populations in the cotton-growing areas of the USA, Egypt and Israel for decades [3]. Recently, resistant populations to sex pheromones have been found in cotton fields of Israel, which compromises the use of this control measure [4]. The use of viral pathogens to manage PBW was also investigated. As with other lepidopteran insects, the larvae of PBW are susceptible to nuclear polyhedrosis viruses (baculoviruses). For instance, *Autographa californica* *nuclear phlyhedrosis virus* (AcNPV) was tested in bait formulation against PBW larvae, which resulted in significantly decreased numbers of larvae and thus boll damage [5,6].

Viral pathogens of PBW were previously recorded. The infectivity, symptomatology, histopathology and transmission of AcNPV in pink bollworm larvae were investigated [7]. In addition to the DNA virus, a small RNA virus (picoRNA-like virus) infecting pink bollworm larvae and adults and causing disease symptoms and even death of the infected larvae was isolated from cotton fields of Egypt. This virus infected midgut cells and could be vertically transmitted from infected adults to offspring [8]. However, genomic sequences of the RNA virus have not been reported.

Assembly of high-throughput sequencing data provides a powerful methodology for discovery of viral sequences from insects [9]. Analysis of the assembled transcripts to identify novel viruses is now a common tool for determination of viral sequences and for construction of full-length viral genomes of RNA viruses. Furthermore, viral sequence analysis from the transcriptome often identified multiple viruses that co-infected host insects without causing disease symptoms [10]. Due to the difference in the sex pheromone ratio between laboratory and field populations, and since there are no significantly different expressed genes involved in sex pheromone biosynthesis [4], we attempted to investigate transcriptome variations in viruses in the pheromone glands. Here, we observed significant numbers of contigs that hit viral sequences by BLAST annotations. Through analysis of the putative viral sequences, we identified putative viral sequences representing possible novel viral species, and we herein describe their unique features.

## 2. Materials and Methods

### 2.1. Insect Collection and Pheromone Gland Extraction

Two different populations of pink bollworm were collected in Israel. One was from a laboratory colony (Lab) maintained at the Volcani Institute, Bet-Dagan, Israel, and the other one was collected from a cotton field (Field) near Ein Shemer, Israel, where mating disruption failed [4]. The laboratory population was maintained in the lab for many years but with the periodical addition of field-caught insects from Israel that had never been exposed to the PBW pheromone. The field insects originated from a cotton field in Israel and were reared in the lab to increase the population. Pheromone glands along with ovipositors from adult females of the two populations were removed, immediately placed in RNAlater (Invitrogen), frozen at −80 °C and then shipped to the lab in the United States. 

### 2.2. RNA Isolation and Illumina Sequencing

Methods for RNA isolation, sequencing library preparation and Illumina sequencing were described previously [4]. Briefly, total RNA from 30 pheromone glands was isolated using TRIzol reagent (Invitrogen, Carlsbad, CA, USA) according to the manufacturer’s protocol. A 2100 Bioanalyzer (Agilent Technologies) was used to check the quantity of RNA. After removing rRNA, the mRNA library was prepared and sequenced on an Illumina HiSeq 3000 platform by the DNA facility at Iowa State University, Ames, IA, USA. The stranded total RNA-Seq libraries were sequenced with 150 paired ends and the mRNA library with 100 single reads. All sequencing reads were submitted to the SRA of NCBI under the accession number “SRP140160”. The total number of reads generated from the mRNA library was 3.38 × 10^8^ and 3.35 × 10^8^ in lab and field populations, while the stranded total RNA-Seq libraries were 8.14 × 10^7^, 5.55 × 10^7^ and 4.88 × 10^7^ in the lab population and 9.91 × 10^7^, 6.38 × 10^7^ and 4.86 × 10^7^ in the field population.

### 2.3. De Novo Assembly of Short Reads and Gene Annotation

The quality of all raw reads was checked using FastQC (Babraham Bioinformatics, Cambridge, UK). The de novo assembly was carried out with the short reads assembling program Trinity [11]. To search potential RNA sequences derived from viruses, the assembled contigs were scanned for candidate sequences by BLASTx (E-value < 0.01) in a search against a local virus sequence database, which was organized from NCBI viral sequences (https://www.ncbi.nlm.nih.gov/labs/virus/vssi/#/virus? (1 July 2015)). Contigs that hit viral sequences were selected and further annotated by using the BLASTx program against the NCBI non-redundant (*nr*) protein databases. Details of the viral sequence search and determination were previously described [12]. Viral sequence mapping was performed by using a Perl script, which only mapped perfectly matched reads to the viral sequences [12].

### 2.4. Sequence and Phylogenetic Analysis

The conserved and functional domains of the predicted viral proteins were identified by the Conserved Domain Database (CDD) in NCBI (https://www.ncbi.nlm.nih.gov/Structure/cdd/wrpsb.cgi (accessed on 14 June 2021) and InterProtScan (https://www.ebi.ac.uk/interpro/search/sequence-search (accessed on 14 June 2021). The RNA-dependent RNA polymerase domain of picorna-like viruses was determined by the online NCBI conserved domain search engine (https://www.ncbi.nlm.nih.gov/Structure/cdd/wrpsb.cgi? (accessed on 14 June 2021) with an E-value of <0.001. The identified RdRP sequences were extracted and used for phylogenetic analysis. Multiple sequence alignments of the core motifs were conducted using CLC genomics Workbench 9.5. For the phylogenetic analysis, the sequences were aligned using the ClustalW method. MEGA 7.0 [13] was used for tree construction using the neighbor-joining method with 1000 bootstrap replicates, the Poisson model and pairwise gap deletion options.

## 3. Results

### 3.1. Identification of Sequences Derived from Putative Viruses

The initial screen of potential virus-related sequences by BLASTx against the local viral databases resulted in the selection of ~1% of contigs for further analysis. The selected contigs were further annotated by a BLAST search of the NCBI nr protein databases. The contigs of putative viral origins with a nucleotide length longer than 500 and an amino acid length longer than 100 were selected. In total, there were 19 transcript hits to viruses in the lab population and 21 hits (length > 500 nt) to viruses in the field population (Table 1). Out of the viral sequences found, there were 9 and 11 transcripts of dsDNA viruses in the lab and field populations, respectively, and 8 were homologous to ssRNA viruses in both populations, with 2 of them having positive-sense and 8 negative-sense RNA genomes (Tables S1 and S2). Among those sequences, two viral contigs derived from (+ss)RNA viruses encoded entire viral genes and contained 5′- and 3′-untranslated regions (UTR); hence, these sequences were near full-length virial genomes. There were eight contigs with (−ss)RNA viral sequences that also encoded complete viral genes and hit two (−ss)RNA viruses. Those contigs were also likely derived from viral genomes. In addition, two putative viral transcripts encoding protein sequences were found that hit the RdRp of Formica fusca virus 1 (Contig 1 in lab population and Contig 18 in field population); however, the encoded viral protein appeared truncated. Hence, these two contigs were likely derived from viral sequences integrated in the host genomes. Based on the viral genome structure and BLAST search results, these eight viral RNAs were likely derived from two (+ss) and six (−ss) RNA viruses. We then named the two (+ss)RNA viruses as Pectinophora gossypiella virus1 (PecgV1) and Pectinophora gossypiella virus 4 (PecgV4), and two (−ss)RNA viruses, Pectinophora gossypiella virus 2 (PecgV2) and Pectinophora gossypiella virus 3 (PecgV3). The sequences of these eight ssRNAs were submitted to GenBank under “MN164617 to MN164623 and MZ361082 and MZ361083”. The other two (−ss)RNA viruses belong to *Mononegavirales*.

Amino acid alignment of the six ssRNA viruses between the lab and field populations showed 99% to 100% sequence identity (Table 2). To assess the relative amount of viral RNA in the field and lab populations, we mapped the sequencing reads to the genomes of the RNA viruses. Remarkably, the two (+) ssRNA viruses accumulated very high viral sequences. Totals of 3.34% and 1.09% of the reads were derived from PecgV1 in field and lab populations, respectively, which are equivalent to 39,250 and 4052 folds of sequence coverages. Sequencing reads derived from PecgV4 also contributed to 1.1% and 2.5% of the total reads. The base coverage of PecgV1 was up to 8475-fold for the field sample and 5511-fold for the lab sample. Accumulations of the viral RNA from PecgV2 and PecgV3 were much lower comparing to the two positive RNA viruses. However, the viral sequence coverages of these two viruses were variable between 50- and 710-fold (Table 2). These results indicate that PecgV1 and PecgV4 were highly accumulated in the pheromone gland tissues.

In the NCBI/SRA database, we found transcriptomic sequencing data from larval PBW isolated from a USA population (SRA: SRP062867) [14]. We assembled the transcriptome reads, and only one transcript of 10,031 nt was annotated as a putative viral sequence. Sequence annotation showed the transcript to share 99% sequence identity to PecgV1, indicating that this transcript was derived from PecgV1. Hence, PecgV1 is likely distributed widely in PBW populations, while PecgV2, PecgV3 and PecgV4 were only found in the Israeli populations.

### 3.2. Analysis of Positive-Sense Single-Stranded RNA Viruses

**Figure 1:**
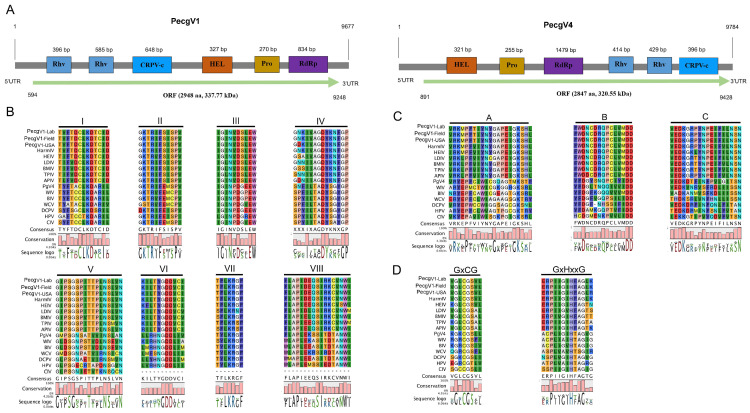
Genome organization and amino acid alignment of PecgV1 and PecgV4. (**A**): CDD and InterProScan analysis of genome structure of PecgV1 and PecgV4. Rhv: Picornavirus capsid protein domain_like; CRPV_c: CRPV capsid protein like; Hel: RNA helicase; Pro: protease; RdRp: RNA-dependent RNA polymerase; UTR: untranslated region; ORF: open reading frame. (**B**): RdRp amino acid alignment. (**C**): Helicase amino acid alignment. (**D**): Protease amino acid alignment.

BLASTp analysis of the amino acid sequences indicated that PecgV1 was a member of *Iflaviridae*. Although protein annotation of PecgV4 showed a close relationship to iflaviruses, its genome orientation is different from that of an iflavirus. For example, the structural and non-structural proteins are arranged in the opposite position in PecgV4 (Figure 1). This picorna-like virus was observed in recently discovered unclassified insect picorna-like viruses, Diaphorina citir picorna-like virus (DcPLV) (KT698837.1) [15], Riptortus pedestris virus-2 (RpV2) (MN078225.1) and Hubei picorna-like virus 33 (HPLV33) (NC_033210.1) [16]. The replication-related L-fragment of PecgV2 and PecgV3 hit the families of *Phasmaviridae* and *Phenuiviridae*, respectively.

The PecgV1 polyprotein suggested that PecgV1 is highly homologous to an iflavirus (Helicoverpa armigera iflavirus) isolated from Helicoverpa armigera with 78% identity (Tables S1 and S2), which represents a possible new species in *Iflaviridae*. The genome of 9677 nt comprised a 593-nt 5′ UTR with a putative internal ribosome entry site (IRES), a single ORF polyprotein of 2948 aa with a predicted molecular weight of 337.77 kDa and a 429-nt 3′ UTR followed by a poly A tail (Figure 1A). PecgV4 is 9784 nt in length and also encoded a polyprotein of 2846 aa with a predicted molecular weight of 320.55 kDa. It is notable that PecgV4 has a different protein orientation comparing to iflaviruses in which its capsid protein domains were located in the C’-terminus (Figure 1C). The structural and non-structural protein domains of the PecgV4 polyprotein showed ~40% identity to the counterparts of the polyprotein of Lysiphlebus fabarum rna-virus type A, which is an iflavirus. The other top hits of the PecgV4 polyprotein were also from Iflaviridae.

Despite the differences in polyprotein structures of PecgV1 and PecgV4, the conserved protein domains identified from PecgV1 and PecgV4 were similar (Figure 1A), including the RNA helicase (pfam00910), protease (peptidase_C3G super family) (cl13774) and RdRp (cd01699) domains in the non-structural polyprotein half, and two Rhv-like (cd00205) and a CrPV-coat capsid (cl07393) domains in the structural polyprotein half. All of these domains are common in iflaviruses. Further analysis identified eight picornaviral conserved motifs of RdRP domains (Figure 1B), three conserved motifs from RNA helicase domains (Figure 1C) and two conserved protease motifs (Figure 1D) from PecgV1 and PecgV4. These motifs were previously described by Koonin et al. [17]. Sequence comparison of the motifs among PecgV1 and several iflaviruses from Helicoverpa armigera, Bombyx mori, Lymantria dispar, Antheraea pernyi and Thaumetopoea pityocampa showed highly conserved amino acid compositions in those motifs (Figure 1). In contrast, however, the amino acid motifs of PecgV4 were more divergent compared to those of PecgV1 (Figure 1).

**Figure 2:**
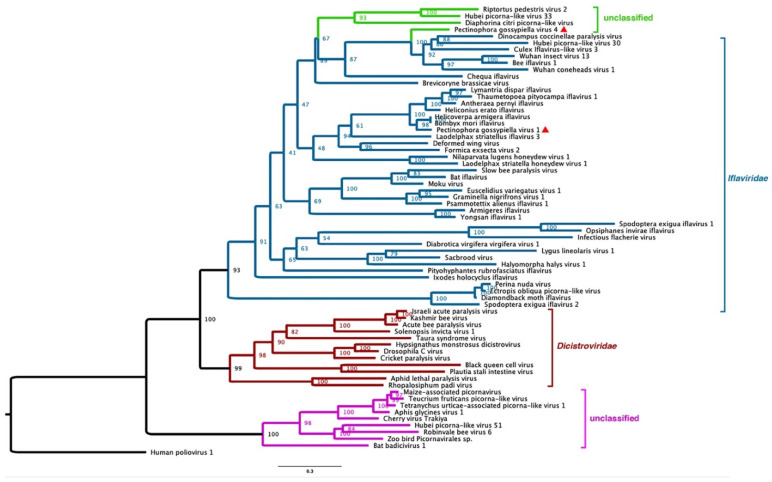
Phylogenetic tree of PecgV1 and PecgV4 (red triangle) with other iflaviruses. RdRP domain sequences (cd) were used. The accession numbers for sequences are listed in File S1. The amino acid sequences were aligned using ClastalW, and then the tree was constructed using Mega 7.0 with neighbor-joining method and evaluated with 1000 bootstrap replicates.

Phylogenetic analysis of RdRP domains of PecgV1 and PecgV4 with known iflaviruses, dicistroviruses and other unclassified picorna-like viruses revealed that PecgV1 is closely related to the viruses isolated from *H. armigera* and *B. mori*. The three iflaviruses together with three lepitopteran iflaviruses form a subclade (Figure 2). Although PecgV4 is structurally similar to DcPLV, HPLV33 and RpV2, the RdRP of PecgV4 is grouped with several unclassified insect viruses with iflaviral genome structures (Figure 2); phylogenetically, PecgV4 is more closely related to iflaviruses than to the viruses which have the same genome structures as PecgV4.

### 3.3. Negative-Sense Single-Stranded RNA Viruses

**Figure 3:**
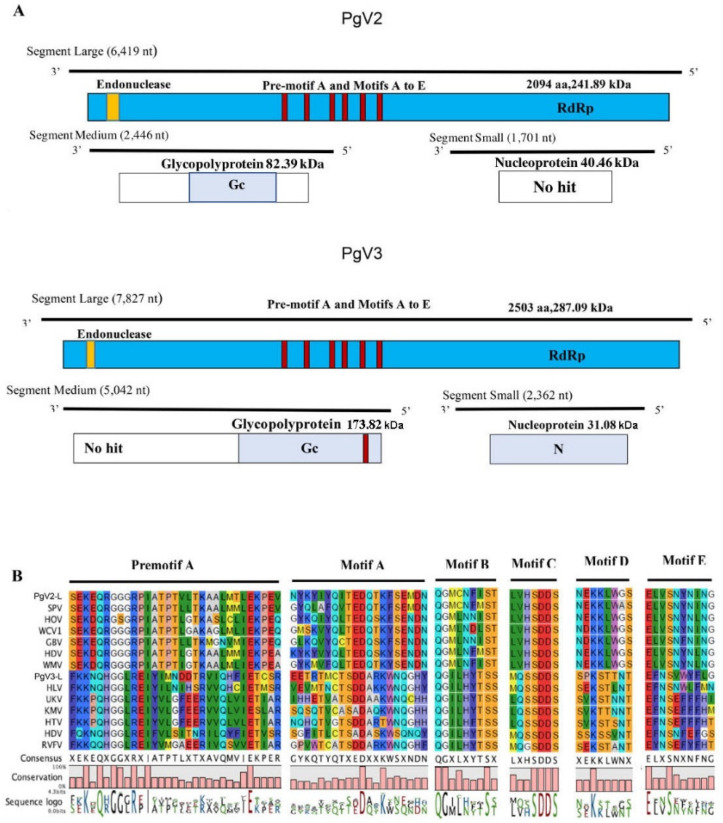
Genome organization and amino acid alignment of PecgV2 and PecgV3. (**A**): CDD and InterProScan analysis of genome structure of PecgV2 (top) and PecgV3 (below). (**B**): Amino acid alignment between conserved RdRp premotif A and motifs A–E of PecgV2 and PecgV3, and selected (−ss)RNA viruses. The accession numbers of sequences for amino acid alignment are listed in File S1; red bars represent the conserved motifs in the genome.

The initial annotation of PecgV2 and PecgV3 suggested that these two viruses were (-ss)RNA viruses belonging to *Bunyavirale*. The genomes of bunyaviruses consist of three subgenomic RNA segments: large, medium and small (L, M and S). The L segment encodes RdRP, and M and S encode glycoproteins and nucleocapsid proteins, respectively [18]. For PecgV2, the large segment was 6419 nt in length, which contained a complete CDS of RdRp. The RdRp of PecgV2 is 2094 aa with a predicted molecular weight of 241.89 kDa (Figure 3A). A Bunya_RdRp domain (pfam0419, interval 618–1231aa) was determined by CDD analysis with an E-value of 8.09 × 10^−7^. BLAST analysis of the RdRP protein of PecgV2 showed a high amino acid identity (62%) to that of Seattle prectang virus (SPV), which is a member of the family *Phasmaviridae* and was isolated from the green pug (*Pasiphila retangulata*), a lepidopteran. The M segment of PecgV2 was 2446 nt in length. The top BLASTp hit was 42.47% identity to the glycoprotein of Seattle prectang virus. The conserved domain prediction showed that there is a phlebovirus glycoprotein G2 domain in the middle region (interval: 268–525, E-value: 1.64 × 10^−3^). The S segment of PecgV3 was 1701 nt, encoding a potential nucleoprotein with 40.46 kDa. The BLASTp result showed 56.74% identity to the nucleocapsid of Seattle prectang virus; however, CDD analysis did not find any conserved domains.

The genome of PecgV3, with its three segments, was recovered from the sequencing reads. The three encoded viral proteins hit Hubei lepidoptera virus 1 (HLV1) of *Bunyavirale* by the BLASTp search. The L segment was 7827 nt in length and encoded a putative RdRp of 2503 aa with a predicted molecular weight of 287.09 kDa (Figure 3A). The L segment of PecgV2 and PecgV3 only shared 12% amino acid identity, indicating they are not closely related. The conserved Bunya_RdRp L protein domain was predicted to be between 809 and 1485aa (E-value 3.68 × 10^−74^). The alignment of the RdRp from PecgV2-L and PecgV3-L and other (−ss)RNA viruses indicates that the amino acid sequences of their RdRps consist of the pre-motif A and motifs A through E, which are highly conserved in negative-sense RNA viral polymerases and common to all other *Bunyavirales* characterized to date [19] (Figure 3B). We found three basic residues (K, R and R/K) in promotif A and a glutamic acid (E) downstream of premotif A, which are conserved in bunyaviral RdRps [20,21]. SDD sequences in motif C were also found in both viruses. The other motifs were slightly different between these two viruses. The M segment of PecgV3 was 5042 nt in length and encoded a surface glycoprotein of 1558 aa with a mass of 173.82 kDa. BLASTp results show that the M segment had 27% identity to the putative glycoprotein of HLV1. The conserved domain predicted by CDD showed a single transmembrane region close to the C-terminus of the glycoprotein (interval: 1052–1520, E-value: 1.14 × 10^−15^). The online programs TMHMM and TMPred showed there are two transmembrane domains in the middle region and one transmembrane domain in the C-terminus. This result is expected as multiple internal transmembrane domains are conserved in *Phenuiviridae*. The S segment of PecgV3 is 2362 nt in length and encoded for a unique protein of 276 aa with a predicted mass of 31.08 kDa. Protein annotation showed that it was closely associated with the N protein of members of the genus *Phasivirus*. CDD analysis showed there is a *Tenuivirus*/*Phlebovirus* nucleocapsid protein domain (interval: 23–245, E-value: 3.98 × 10^−6^).

**Figure 4:**
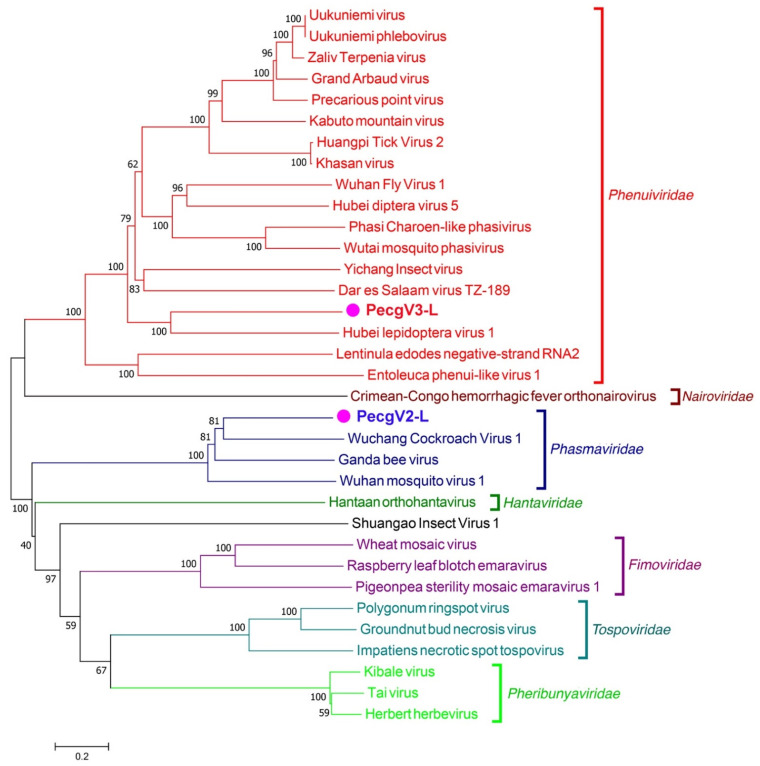
Phylogenetic trees of PecgV2 and PecgV3 with other (−ss)RNA with RdRp sequences. The accession numbers for sequences are shown in File S1. The amino acid sequences were aligned using ClastalW, and then the tree was constructed using Mega 7.0 with neighbor-joining method and evaluated with 1000 bootstrap replicates.

Phylogenetic trees were constructed based on the amino acid sequences of the RdRp segment (Figure 4). The accession numbers of the other viruses are shown in File S1. PecgV2 RdRp was clustered together with the family *Phasmaviridae* in the order *Bunyavirales*. PecgV3 was clustered together with HLV1, a member in the family *Phenuiviridae*. All the data show that PecgV2 and PecgV3 are different viruses and both belong to the order *Bunyavirales*, but in different families.

### 3.4. Endogenous Viral Elements (EVE) Derived from RNA Viruses

In mammals, retroviral envelope genes play an important role in cell–cell fusion during placentation, and they might have anatomic and immunological functions [22]. The two contigs (lab contig 1 and field contig 18; lab contig 17 and field contig 19) that hit viral genes and were annotated as a putative EVE were both derived from mononegaviruses, a viral group consisting of one segment of the (−)ssRNA genome. The initial annotation of lab contig 1/field contig 18, a 2325-nt RNA fragment, suggested that the encoded CDS was likely derived from a nucleocapsid gene (N) of a virus close to Spodoptera frguiperda rhabdovirus. Further analysis by BLASTp against nr databases revealed that the encoded protein is a homolog of uncharacterized proteins from *Ostrinia furnacalis*, *Papilio* spp., *Aphantopus hyperantus* and other insects. Lab contig 17/field contig 19 was 4423 nt in length, encoding two CDSs. They both hit RdRP of Formica exsecta virus 4 (FeV4) or Formica exsecta virus 1 (FeV1)*,* members of *Monoegavirales*. A full-length RdRP of FeV4 or FeV1 is 1877aa; hence, the two CDS of the contigs are actually truncated sequences. In addition, no sequences of other viral genes related to the RdRPs were assembled from the reads, and these results suggest that the RdRP hit contigs were putatively derived from the EVE of the host.

## 4. Discussion

New viruses are difficult to discover with the traditional viral detection methods because they require isolation of viruses followed by whole genome sequencing. However, high-throughput next-generation sequencing technologies and bioinformatics have been applied to virus discovery in various organisms, including humans [23,24], arthropods [25,26,27], plants [28,29] and fungi [30,31,32]. Transcriptome sequencing and deep sequencing of viral small RNA analysis have also been used for the discovery of viruses in plant and insect hosts [33,34].

The pink bollworm, as a pest species, quickly spread around the world as it was first reported on infested cotton in Egypt in 1913 [35] and in 1917 in the USA [36]. In the present study, through transcriptome analysis, we discovered novel RNA viral sequences likely derived from viruses infecting the pink bollworm and derived from mononegaviruses (EVE). The viral sequences were not different between the lab and field populations from Israel. The four viruses share 99% to 100% sequence identity in the comparison of lab and field populations. Higher viral sequence coverages from the sequencing reads indicate that they are consistently expressed in the pink bollworm. However, fewer viral sequences were recovered from the American larval sample analyzed by Tassone et al. [14]. Only PecgV1 was observed in both the Israeli and American samples. The transcriptome of the Israeli population was from the pheromone gland with ovipositors, while the USA population was from the midgut of larvae. We assume that PecgV1 is continuously infecting PBW in different development stages, at least in adults and larvae, and it is passed to the next generation vertically. The fewer viral sequences in the American samples might be due to the lower sequence coverage in the larva RNA sample, different insect stages or various other potential factors. It is interesting that extremely high accumulations of the viral RNA derived from PecgV1 and PecgV4 were found in the pheromone glands, suggesting that these viruses replicated at high levels in adult pink bollworms. However, no visible disease symptoms were observed in the adults. Hence, the potential biological functions of the viruses to the hosts remain to be investigated as well as the tissue distribution within the host.

To date, four genomic types of picornaviruses isolated from insects have been identified. These viruses share similar conserved domains: the RdRp (cd0169) and Rh-V domains and other motifs. Members of *Iflaviridae* and *Dicistroviridae* could cause serious disease symptoms to the host insect. For instance, *Aphid lethal paralysis virus* (ALPV), a dicistrovirus, could significantly reduce aphid populations [37,38]. Dicistroviruses have two ORFs: ORF1 encodes non-structural polyproteins, while ORF2 expresses structural proteins. The other commonly found picorna-like viruses are iflaviruses. The genome of iflaviruses only expresses a single ORF, encoding the non-structural proteins (NSPs); the structural proteins (SPs) are encoded in one ORF [39]. The NSPs are located in the N-terminal of the protein sequence, while the C-terminal contains SPs, and these are one of the genomic characteristics of iflaviruses. Recently, two new types of unclassified insect-infecting picorna-like viruses were discovered by analyzing assembled transcripts form transcriptome sequencing data. Aphis glycines virus 1 (Accession KM013260.2) represents a new group of dicistro-like viruses, in which the genome encodes two ORFs, but is different from dicistroviruses in that the SPs are located in the N-terminal and the NSPs are in the C-terminal half of the protein sequence. PecgV4, DcPLV, HPLV33 and RpV2 are members of a group of picorna-like viruses, whose genomes encode a single ORF with NSP in the N-terminal half and SP in the C-terminal half of the protein sequence. Phylogenetic analysis of RdRp domains indicated that PecgV1 and the viruses with PecgV4-like genomes are close to iflaviruses, but distant from dicistroviruses (Figure. 2), suggesting that the two groups of picorna-like viruses which encode only a single ORF might have evolved from the same ancestors, although the NSP and SP are in opposite locations for the two groups of viruses, while the two groups of viruses in which the NSP and SP are independently encoded were closely related in viral evolution. The phylogenetic analysis based on the RdRp domains showed that the unclassified dicistro-like viruses appeared earlier in the phylogeny, suggesting that the other picorna-like viral groups might have evolved from the newly discovered dicistro-like viruses (Figure 2).

PecgV2 and PecgV3 were defined as (-ss)RNA viruses. Based on the amino acid alignment and phylogenetic analysis, it is clear that the sequences of PecgV2 and PecgV3 were derived from different viruses. PecgV2 is a virus in the family *Phasmaviridae.* Viruses of this family are known to be isolated from insect hosts, while PecgV3 relates to viruses in the family *Phenuiviridae*, which infect both vertebrates and invertebrates. Hence, the pink bollworm is likely the host of PecgV2 and PecgV3. It has been proposed that plant negative RNA viruses evolved via horizontal virus transfer from vertebrates and mediated by arthropod vectors. Therefore, it is reasonable that members of *Bunyavirales*, mostly detected in plants and vertebrates, can be found in invertebrates, such as the pink bollworm in this study and other insects. The ancestors of plant and vertebrate negative RNA viruses are thought to have evolved from a natural reservoir which is represented by invertebrates via horizontal virus transfer, indicating that diverse invertebrates show similar viruses in the same ecological niche [40]. Investigation of the viruses in other insects would help to give a strong support of this view.

EVE sequences were commonly observed in insect genomes [41]. In addition to retroviral-related EVEs, EVEs derived from DNA and RNA viruses and (+) ssRNA, (-) ssRNA and double-stranded (ds)RNA viruses were observed in the same insect species. Most of the EVE sequences were disrupted in the host genomes [12]. Rhabdovirus-like EVE was previously observed in *S. frugiperda* cells [42]. Rhabdorivirus EVE derived from Spodoptera frugiperda rhabdovirus was also observed in RNA-seq of *S. frugiperda* larvae (Liu, unpublished results). We identified two transcripts likely derived from EVEs of unknown rhabdoviruses. The transcript encoding the N gene also hit other uncharacterized genes of several lepidopterans (data not shown), suggesting that the EVEs of mononegaviruses are common in these insects. Recovery of transcripts of the EVEs suggested that the EVEs were active. However, the coding regions of the contigs encoded two truncated RdRp sequences, indicating that the partial RdRp might not have biological functions. Some evidence has pointed to RNA-level functions for insect nonretrovial EVEs [43,44], but whether the EVE transcripts were translated and have functions in PBW needs to be further investigated.

In this study, we found the PBWs were infected by multiple RNA viruses. Picorna-like virus isolated from the pink bollworm population of Egypt was previously reported [8]. An Egyptian isolate of picorna-like virus caused severe pathogenetic impacts on the insects, specifically infection of the virus, resulting in death of the diseased larvae [8]. It is not clear whether the presently identified PecgV1 and PecgV4 were the same viruses as the viruses reported in 1995. Infection with picorna-like viruses does not always cause disease symptoms in the host insect. The dicistrovirus ALPV has been isolated from many insect species but was only observed causing diseases in the field population of cherry oat aphids (*Rhopasiphum padi*) [37,38]. However, it was asymptomatic in isolates from laboratory populations of pea aphids (*Acyrthosiphon pisum*) and *R. padi* [45]. Hence, it is possible that PecgV1 or PecgV4 may be an isolate of the previously observed picorna-like virus in Egypt. Our research provides the viral sequences that have potential for development of novel biological control agents for management of this worldwide devastating cotton insect pest.

## Figures and Tables

**Table 1:** Number of viral RNA fragments found in the transcriptome of pheromone gland in two populations.

	Virus	dsDNA Virus	ssRNA Virus	
			Positive-Sense	Negative-Sense
Lab	19	9	2	8
Field	21	11	2	8

**Table 2:** Comparison of ssRNA viruses between two populations of the PBW.

ssRNA Viruses	AA Length	Identity (Field vs. Lab)	Sequence Coverage (Field/Lab)	% of Reads (Field/Lab)
PecgV1	2948	99%	39,250.6/4052	3.34/1.09
PecgV2-L	2093	99%	87.4/56	0/0
PecgV2-M	724	99%	49.3/168.9	0/0
PecgV2-S	364	99%	286.7/487.3	0/0
PecgV3-S	277	100%	106.1/50	0/0
PecgV3-M	1558	99%	170.8/103.2	0/0
PecgV3-L	2502	99%	709.9/632.4	0.05/0.14
PecgV4	2846	99%	8474.6/5510.7	1.1/2.25

## Data Availability

All sequencing reads were submitted to the SRA of NCBI under the accession number “SRP140160”.

## Supplementary Materials

The following are available online at https://www.mdpi.com/article/10.3390/insects12060556/s1, Table S1: Viruses found in the lab population, Table S2: Viruses found in the field population, File S1: Full name and accession numbers of viruses in sequence alignment and phylogenetic analysis.
