# Supplementary material for: Novel RNA Viruses from the Transcriptome of Pheromone Glands in the Pink Bollworm Moth, Pectinophora gossypiella
Source: Insects. 2021 Jun 15;12(6):556. doi: 10.3390/insects12060556 (PMC8232680; doi:10.3390/insects12060556)
Supplement: Supplementary file 1 [file insects-12-00556-s001.zip › S1 Table_revised.pdf]

S1 Table. Viruses found in Lab population

Lab population	nt	aa	Full?	GenBank description	Identity (%)	Access number	Type	Potential source
Contig1	2153	345	N	L polymerase RdRp [Formica fusca virus 1]	32.83	AYW51538	(-ss)RNA	Host genome
Contig2 (PecgV1)	9677	2948	Y	polyprotein [Helicoverpa armigera iflavirus]	78.25	YP_009344960	(+ss)RNA	Virus
Contig3 (PecgV2)	6419	2093	Y	RNA-dependent RNA polymerase [Seattle Prectang virus]	62.05	AOF41423	(-ss)RNA	Virus
Contig4 (PecgV3-L)	7827	2502	Y	RNA-dependent RNA polymerase [Hubei lepidoptera virus 1]	33.26	YP_009330283	(-ss)RNA	Virus
Contig5 (PecgV3-S)	2362	276	Y	putative nucleoprotein [Hubei lepidoptera virus 1]	31.49	YP_009330256	(-ss)RNA	Virus
Contig6 (PecgV3-M)	5042	1558	Y	putative glycoprotein [Hubei lepidoptera virus 1]	27.73	YP_009330257	(-ss)RNA	Virus
Contig7 (PecgV4)	9782	2846	Y	hypothetical protein [Wuhan insect virus 13]	37.36	YP_009342321	(+ss)RNA	Virus
Contig8	1009	259	Y	hypothetical protein B5V51_1641 [Heliothis virescens]	34.46	PCG71644	dsDNA	Host genome
Contig9	2059	256	Y	orf10-like protein [Peridroma alphabaculovirus]	30.4	YP_009049835	dsDNA	Host genome
Contig10	1784	437	Y	ORF61 [Xestia c-nigrum granulovirus]	27.89	NP_059209	dsDNA	Host genome
Contig11	1439	257	Y	orf10-like protein [Peridroma alphabaculovirus]	27.73	YP_009049835	dsDNA	Host genome
Contig12	765	170	Y	unknown similar to MacoNPV-B orf57 [Choristoneura biennis entomopoxvirus]	31.34	YP_008004381	dsDNA	Host genome
Contig13	791	231	N	cathepsin-like protein [Helicoverpa armigera nucleopolyhedrovirus]	50.45	AIY24949	dsDNA	Virus
Contig14	758	130	N	cathepsin-like cysteine proteinase [Spodoptera litura nucleopolyhedrovirus]	46.88	NP_258322	dsDNA	Virus
Contig15	1056	144	N	cathepsin [Helicoverpa armigera nucleopolyhedrovirus G4]	46.81	NP_075125	dsDNA	Virus
Contig16	1249	353	N	cathepsin [Choristoneura fumiferana multiple nucleopolyhedrovirus]	47.57	XP_028167656	dsDNA	Unknown
Contig17	1811	552	N	uncharacterized protein LOC114355158 [Ostrinia furnacalis]	37.45	XP_028163655	(-ss) RNA	Host genome
Contig 18 (PecgV2-M)	2446	724	Y	glycoprotein precursor [Seattle Prectang virus]	42.47	YP_009666958	(-ss) RNA	Virus

Contig 19 (PecgV2-S)	1701	252	Y	nucleocapsid [Seattle Prectang virus]	56.74	YP_009666960	(-ss) RNA	Virus
