# Supplementary material for: Novel RNA Viruses from the Transcriptome of Pheromone Glands in the Pink Bollworm Moth, Pectinophora gossypiella
Source: Insects. 2021 Jun 15;12(6):556. doi: 10.3390/insects12060556 (PMC8232680; doi:10.3390/insects12060556)
Supplement: Supplementary file 1 [file insects-12-00556-s001.zip › S2 Table_revised.pdf]

S2 Table. Viruses found in Field population

Field Population	nt	aa	Full ?	GenBank description	Identit y(%)	Access	Type	Potential sourse
Contig1	754	189	N	unknown similar to MacoNPV-B orf57 [Choristoneura biennis entomopoxvirus]	30.88	YP_008 004381	dsDNA	Unknown
Contig2	861	136	N	cathepsin-like cysteine proteinase [Spodoptera litura nucleopolyhedrovirus]	46.62	NP_258 322	dsDNA	Unknown
Contig3	593	191	N	transposase mut [Lambdina fiscellaria nucleopolyhedrovirus]	49.73	YP_009 133324	dsDNA	Unknown
Contig4	1288	333	N	zingipain-2-like [Ostrinia furnacalis]	46.25	XP_028 167656	dsDNA	Unknown
Contig5	776	226	N	cathepsin-like protein [Helicoverpa armigera nucleopolyhedrovirus]	50.45	AIY249 49	dsDNA	Unknown
Contig6	1390	376	N	hypothetical protein [Samia ricini nucleopolyhedrovirus]	33.67	BBD51 232	dsDNA	Unknown
Contig7	631	164	N	Mabr_orf10 [Mamestra brassicae multiple nucleopolyhedrovirus]	32.7	AFP957 29	dsDNA	Unknown
Contig8 (PecgV1)	10063	2948	N	polyprotein [Helicoverpa armigera iflavirus]	79	YP_009 344960	(+ss)RNA	Virus
Contig9 (PecgV2)	6423	2093	Y	RNA-dependent RNA polymerase [Seattle Prectang virus]	62	AOF41 423	(-ss)RNA	Virus
Contig10 (PecgV3-S)	2352	276	Y	putative nucleoprotein [Hubei lepidoptera virus 1]	31.49	YP_009 330256	(-ss)RNA	Virus
Contig11 (PecgV3-M)	5115	1559	Y	putative glycoprotein [Hubei lepidoptera virus 1]	26.86	YP_009 330257	(-ss)RNA	Virus
Contig12 (PecgV3-L)	7762	2502	Y	RNA-dependent RNA polymerase [Hubei lepidoptera virus 1]	35	YP_009 330283	(-ss)RNA	Virus
Contig13 (PecgV4)	9711	2846	N	hypothetical protein [Wuhan insect virus 13]	37	YP_009 342321	(+ss)RNA	Virus
Contig14	948	257	Y	orf10-like protein [Peridroma alphabaculovirus]	28	YP_009 049835	dsDNA	Host genome
Contig15	2164	256	Y	unknown [Antheraea pernyi nucleopolyhedrovirus]	27	ABQ12 247	dsDNA	Host genome
Contig16	1710	437	Y	ORF61 [Xestia c-nigrum granulovirus]	27	NP_059 209	dsDNA	Host genome
Contig17	2215	204	Y	transposase mut [Lambdina fiscellaria nucleopolyhedrovirus]	46	YP_009 133324	dsDNA	Host genome
Contig18	4219	574	N	L polymerase RdRp [Formica fusca virus 1]	52	AYW5 1538	(-ss)RNA	Virus
Contig 19	2325	532	Y	uncharacterized protein LOC114355158 [Ostrinia furnacalis]	37.8	XP_028 163655	(-ss)RNA	Host genome

Contig 20 (PecgV2-M)	2489	725	Y	glycoprotein precursor [Seattle Prectang virus]	42.47	YP_009 666958	(-ss)RNA	Virus
Contig 21 (PecgV2-S)	1715	365	Y	nucleocapsid [Seattle Prectang virus]	56.74	YP_009 666960	(-ss)RNA	Virus
